# Impact of Diabetes on Short-Term and Long-Term Outcomes of Ampullary Adenocarcinoma Patients after Curative Pancreatoduodenectomy

**DOI:** 10.3390/curroncol29100528

**Published:** 2022-09-20

**Authors:** Xiaojie Zhang, Chongyuan Sun, He Fei, Zefeng Li, Chunguang Guo, Yingtai Chen, Xu Che, Dongbing Zhao

**Affiliations:** 1 Department of Pancreatic and Gastric Surgical Oncology, National Cancer Center/National Clinical Research for Cancer/Cancer Hospital, Chinese Academy of Medical Sciences and Peking Union Medical College, Beijing 100021, China; 2 Department of Hepatobiliary and Pancreatic Surgery, National Cancer Center/National Clinical Research Center for Cancer/Cancer Hospital & Shenzhen Hospital, Chinese Academy of Medical Sciences and Peking Union Medical College, Shenzhen 518116, China

**Keywords:** ampullary carcinoma, diabetes, postoperative complications, recurrence, survival

## Abstract

Background: Many studies have confirmed that diabetes was associated with prognosis in many malignant cancer types. However, the impact of diabetes on ampullary carcinoma (AC) has not been investigated. Methods: A total of 266 AC patients in the National Cancer Center of China between January 1998 and December 2020 were retrospectively reviewed. The postoperative complication rate, postoperative recurrence rate, and long-term survival were compared between the diabetes group and the no diabetes group. Results: A total of 32 AC patients (12.03%) were diagnosed with diabetes before surgery. In total, 111 patients (41.73%) had one or more postoperative complications, and there was no perioperative death. There was no statistically significant difference regarding postoperative complications between the diabetes group and the no diabetes group. Altogether, 120 patients (45.11%) experienced postoperative recurrence. Multivariate analysis revealed that diabetes was an independent risk factor for the recurrence (OR: 2.384, 95% CI: 1.065–5.336, *p* = 0.035), OS (HR: 1.597, 95% CI: 1.005–2.537, *p* = 0.047), and RFS (HR: 1.768, 95% CI: 1.068–2.925, *p* = 0.027) in AC patients after curative pancreatoduodenectomy. Conclusions: Diabetes may adversely affect the recurrence of patients with AC after curative pancreaticoduodenectomy, leading to an increased risk of poor prognosis in early-stage patients. Further studies involving a large sample size are needed to validate our results.

## 1. Background

Ampullary carcinoma (AC) is a rare malignant tumor with an incidence of 3.8 cases/1,000,000 in men and 2.7/1,000,000 in women, accounting for approximately 0.2% of tumors in the gastrointestinal tract [1,2]. In most cases of AC, patients were diagnosed at an early stage due to the early symptoms [3]. To date, surgical resection remains the potential curative strategy in AC patients [4]. Generally, the prognosis of AC is poor, with a 5-year survival rate of less than 45% in the resected patients [5].

Tumor growth, invasion, and metastasis are all closely related to metabolism [6]. Diabetes is a chronic systemic metabolic disease that affects the major metabolic pathway throughout the body. Several studies have demonstrated that diabetes was strongly associated with tumor progression and survival in various cancer types, including breast cancer [7], pancreatic cancer [8], gallbladder cancer [9], epithelial ovarian cancer [10], esophageal cancer [11,12], colorectal cancer [13,14], and lung cancer [15,16]. However, to the best of our knowledge, no study has investigated the relationship between diabetes and AC. Therefore, the current study aimed to detect the impact of diabetes on short-term and long-term outcomes of ampullary adenocarcinoma patients after curative pancreatoduodenectomy.

## 2. Methods

### 2.1. Patients and Study Design

The study included pathologically confirmed AC patients who underwent curative surgical resection at the National Cancer Center of China between January 1998 and December 2020. The clinicopathologic characteristics were retrospectively reviewed, and the diagnosis of diabetes was based on the medical history. The excluding criteria were listed as follows: (i) patients with other tumor histories; (ii) patients with positive surgical margins; (iii) lymph node resection was not performed or the lymph node resection data were missed; (iv) patients whose critical clinical information was not recorded or missed. Patients were included in the diabetes group only if they had a clear diagnosis of diabetes according to World Health Organization or American Diabetes Association criteria.

All the patients in the current study underwent standard Whipple procedures via laparotomy, and there were no patients who received an operation type of pylorus-preserving pancreaticoduodenectomy. The extended lymphadenectomy was conducted as standard procedure. Finally, a total of 266 patients were enrolled in the study. The requirement of informed consent was waived because this study is an observational, retrospective cohort study.

### 2.2. Covariates and Outcomes

The major covariates in the current study included three parts: (a) basic clinical characteristics, such as sex, age, and preoperative jaundice; (b) pathologic characteristics, such as tumor size, differentiation, tumor stage, and blood vessel invasion; (c) treatment information, such as intraoperative transfusion, lymph nodes resection, and adjuvant treatment. The diagnosis of the postoperative gastroenteric anastomotic fistula was made according to the upper digestive tract iodine and various clinical manifestations such as fever, abdominal pain, and peritonitis. The postoperative pancreatic fistula was diagnosed in accordance with the guidelines of the International Study Group of Pancreatic Fistula. The postoperative biliary fistula was defined as the detection of bile components from peritoneal drainage.

Postoperative follow-up data were collected through telephone reviews, outpatient follow-up, and the death registry system. All the patients were followed up until December 2020, and the median follow-up time was 34 months (ranges: 1~240 months).

The short-term outcomes include surgical time, perioperative blood transfusion, and major postoperative complications. The long-term outcomes include postoperative recurrence status, recurrence-free survival time (RFS), and overall survival time (OS). Recurrence and metastasis were confirmed through postoperative imaging examination, including contrast-enhanced computer tomography CT scan, magnetic resonance imaging (MRI), and positron emission tomography CT (PET-CT). According to the recurrence sites, the initial recurrence was divided into locoregional recurrence and systemic recurrence. The RFS was defined as the time from the surgery to the recurrence. The OS was defined as the time from surgery to death for any reason.

### 2.3. Statistical Analysis

All the data analysis was performed with IBM SPSS Statistics for Windows, Version 22.0 (IBM Corporation, Armonk, NY, USA). In order to explore differences in categorical baseline characteristics between the diabetes group and the no diabetes group, the chi-squared test or Fisher’s exact test was used. Univariate and multivariate Cox proportional hazard regression analyses were conducted to determine the prognostic factors. In addition, univariate and multivariate logistic multiple linear regression models were implemented to evaluate the impact of diabetes on the classification outcomes. The Kaplan–Meier survival curves were depicted using the log-rank test (GraphPad Prism 8.0.2). A difference with *p* < 0.05 is considered statistically significant.

## 3. Results

### 3.1. Patients Characteristics

A total of 266 AC patients were finally enrolled in the study. The baseline characteristics of patients are summarized in Table 1. The median age of the cohort was 58 (interquartile range: 50–64) years. The male-female ratio in the current study was 1.2. Diabetes was diagnosed in 32 AC patients (12.03%) before hospitalization, and all of them had type-2 diabetes. Obstructive jaundice was the predominant clinical manifestation (198 patients, 74.44%). In our cohort, most of the patients (176 patients, 66.17%) were confirmed with negative lymph node metastasis. A total of 93 patients (34.96%) received postoperative chemotherapy or chemoradiotherapy. The main chemotherapy regimens were SOX (S-1 and oxaliplatin), XELOX (capecitabine and oxaliplatin), FOLFOX (5-fluorouracil and oxaliplatin), and GEMOX (gemcitabine and oxaliplatin). The diabetes group had no significant difference in basic clinicopathologic characteristics compared with the no diabetes group, except for the TNM stage (*p* = 0.05).

### 3.2. Short-Term Safety Outcomes

In total, 111 patients (41.73%) had one or more postoperative complications, and there was no perioperative death. The major postoperative complications were fistula (41 patients, 15.41%), infection (40 patients, 15.04%), gastroparesis (29 patients, 10.90%), and postoperative bleeding (24 patients, 9.02%). However, there was no statistically significant difference between the diabetes group and the no diabetes group regarding perioperative blood transfusion, operation time, and postoperative complications. (Table 2).

### 3.3. Long-Term Survival Analysis

#### 3.3.1. Patterns of Treatment Failure

Postoperative recurrence was the main reason for treatment failure in the current study. Altogether, 120 patients (45.11%) experienced postoperative recurrence. Specifically, 55 patients (20.68%) developed locoregional recurrence, and 71 patients (26.69%) developed systemic recurrence. (Table 3) The liver (21.43%) was the most common site of systemic recurrence in AC patients. Multivariate analysis adjusting for lymph node metastasis, blood vessel invasion, T stage, and TNM stage revealed that patients in the diabetes group had higher proportions of recurrence compared with the no diabetes group (*p* = 0.035). (Table 4).

#### 3.3.2. Survival Analysis

**Figure 1:**
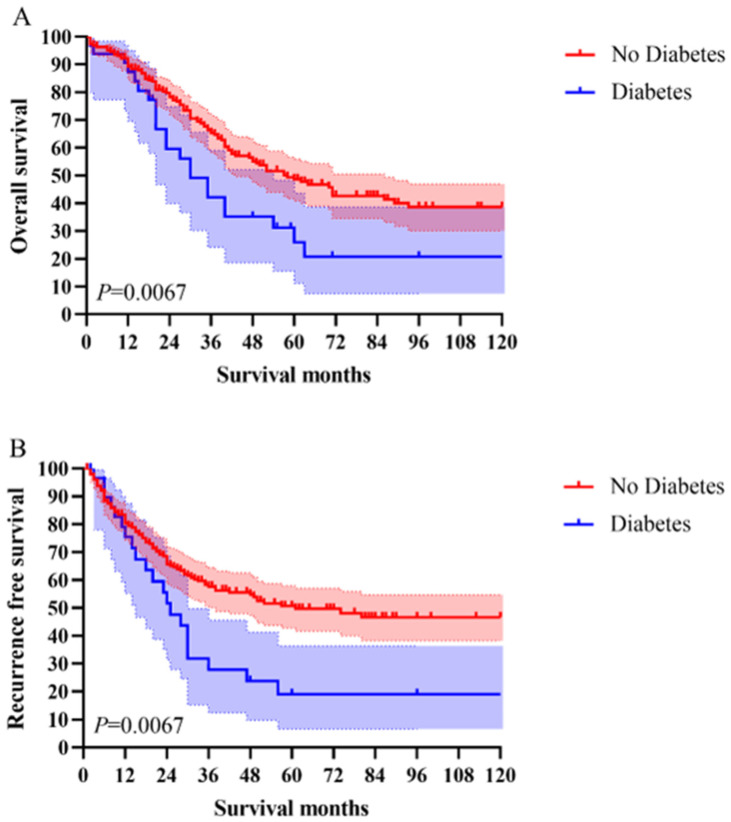
Comparing the survival curves for OS and RFS between the diabetes group and the no diabetes group of AC patients.

**Figure 2:**
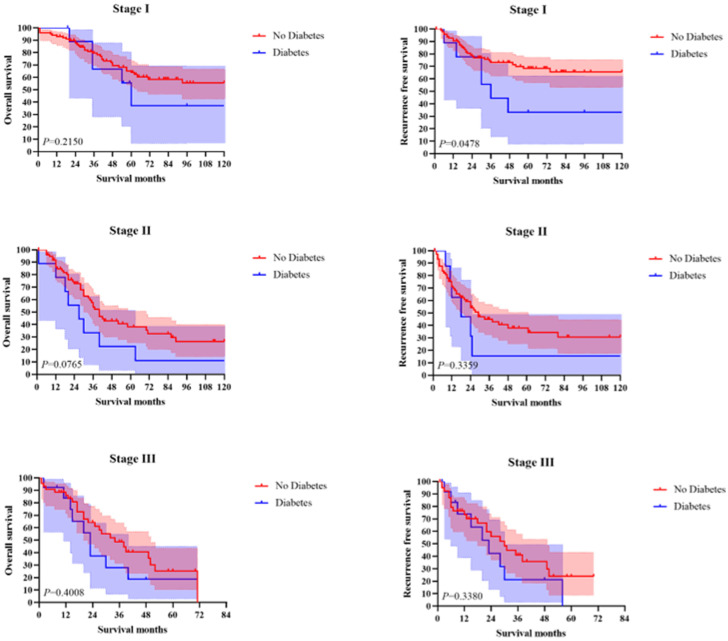
Subgroup survival analysis according to the tumor stage.

The 1-year, 3-year, and 5-year OS in total patients were 86.7%, 60.6%, and 44.4%, respectively. The 1-year, 3-year, and 5-year RFS in total patients were 79.8%, 53.8%, and 46.3%, respectively. The Kaplan–Meier survival curve analysis demonstrated a significant difference in OS and RFS between the diabetes group and the no diabetes group of AC patients (Figure 1). However, in subgroup analysis according to the tumor stage, only in stage I the RFS showed a statistically significant difference (*p* = 0.0478) (Figure 2).

To further investigate the impact of diabetes on survival, we performed univariate and multivariate survival analyses. After performing multivariate adjustments, we found that diabetes was an independent risk factor for the OS (HR: 1.597, 95% CI: 1.005–2.537, *p* = 0.047) and RFS (HR: 1.768, 95% CI: 1.068–2.925, *p* = 0.027) in AC patients after curative pancreatoduodenectomy (Table 4, Table S1 and Table S2).

## 4. Discussions

The impact of diabetes on the short-term and long-term outcomes of digestive tumors is still controversial. To the best of our knowledge, no previous study has been reported about the association between diabetes and outcomes of AC patients who underwent curative pancreaticoduodenectomy. In the current study, we found that diabetes did not increase the risk of postoperative complications in AC patients. However, diabetes might adversely affect the recurrence of patients with AC after curative pancreaticoduodenectomy.

In the present study, we found that diabetes was an independent risk factor for long-term survival in AC patients. The 3-, 5-year OS and RFS in the diabetes group were 42.1%, 26.0%, 27.8%, and 19.1%, while the 3-, 5-year OS and RFS in the no diabetes group were 63.3%, 47.1%, 57.7%, and 50.6%. In addition, we found that diabetes was an independent risk factor for recurrence, especially for systemic recurrence. The similar results were previously reported in the epithelial ovarian cancer [10,17], pancreatic cancer [8,18], breast cancer [7], gallbladder cancer [9], colorectal cancer [13,19,20], and non-small-cell lung cancer [15]. However, the exact mechanism of how diabetes affects the prognosis of AC patients who underwent curative pancreaticoduodenectomy remains unclear.

Previous evidence has demonstrated that diabetic patients have high blood sugar levels, which might correlate with the malignant bioactivity of the tumor cells through various signaling pathways. Firstly, hyperglycemia has been shown to inhibit cellular miR-16 expression, which targets vascular endothelial growth factor (VEGF) signaling, thereby affecting tumor cell proliferation and invasion [21]. In AC, a study found that VEGF expression had some collation with tumor micro-vessel density, which might promote tumor progression and metastasis [22]. Moreover, long-term diabetes may contribute to insulin resistance, which can promote the expression of growth hormone receptors and increase the production of insulin-like growth factor 1 (IGF-1) receptors [11]. Although the IGF-1 signaling pathway in AC is poorly understood, many studies have proved that the IGF-1 signaling pathway could facilitate cancer cell survival [23].

In addition, previous evidence shows that the hyperglycemic state of diabetes may reduce the activity of immune cells, which may have a certain impact on the immune killing of tumor cells [24]. In a German study of 32 patients with esophageal or pancreatic cancer, the results showed that perioperative hyperglycemia reduced postoperative immune function in patients by attenuating postoperative T cells and monocyte function [25]. The impact of these immune function declines may have some impact on the use of postoperative adjuvant therapy, as well as early recurrence and long-term survival.

Moreover, diabetes may affect the prognosis of AC patients in several other potential aspects. Firstly, nutrition is an important part of AC patients after pancreaticoduodenectomy, and nutritional support is of great significance to patients’ tolerance of postoperative adjuvant therapy and long-term survival. However, diabetes may challenge the nutritional structure and nutritional support of patients [12]. Secondly, AC patients with diabetes in the current study were more likely to present with a later stage compared with those without diabetes (*p* = 0.05). Thirdly, a previous study found that perhaps diabetes complications were the main reason for the poor prognosis of colorectal cancer patients rather than diabetes itself by accurately assessing diabetes complications [20]. The results might indicate that prognosis of AC patients who have a prolonged diabetes history is more likely to be affected by diabetes complications.

Notably, although in the overall analysis, our results showed that diabetes was an independent predictor of RFS in AC patients, in the subgroup analysis, this effect was only shown in stage I patients. In addition, patients in the diabetes group had higher tumor stages (40.63% of patients in stage III), which may have contributed to statistical bias. As mentioned above, the impact of diabetes on the prognosis of ampullary cancer patients may be a long-term process. However, for patients with ampullary carcinoma invading surrounding lymph node metastasis, the 5-year survival rate is less than 30% [26]. Thus, diabetes might only have an adverse effect on the prognosis of early-stage AC.

In the present study, we found that diabetes did not increase the risk of postoperative complications or surgical difficulty. However, according to a previous retrospective cohort study, the results demonstrated that diabetes could significantly increase the risk of anastomotic leakage in esophageal squamous cell cancer patients undergoing resection (17.1% vs. 4.3%, *p* < 0.001) [12]. This discrepancy could be due to the following reasons. Firstly, diabetes has the potential to cause microvascular changes that impair responsiveness to congestion [27]. The hyperemia and inflammation of the anastomotic stoma after surgery are important features in promoting tissue healing. Thus, diabetes might affect the ability of the tissue to heal, thereby increasing the risk of anastomotic leakage. Secondly, in the current study, the proportion of AC patients with diabetes was relatively low, which might have introduced bias to the results.

The present study has certain important strengths. Firstly, the current study was the first study focusing on the influence of diabetes on the short-term safety and long-term survival of AC patients who underwent pancreaticoduodenectomy. Secondly, the current study included AC patients with heterogeneous clinicopathologic characteristics over 20 years and long-term follow-up data. Therefore, the findings in the present study provided a foundation for future clinical practice and research. However, we must admit the limitations of the current study. First, this is a retrospective cohort study with a relatively small sample size, and the proportion of AC patients with diabetes was relatively low. Especially when subgroup analysis was performed, the small number of cases may reduce the statistical effect. Second, due to a lack of data, the duration of diabetes and the status of diabetes control could not be further analyzed. Third, the follow-up time of some patients was relatively short, which is a limitation when pushing the results into clinical practice. Fourth, in this retrospective study, patients with diabetes were diagnosed by clinical history. Blood glucose monitoring and management were carried out before surgery for ampullary carcinoma. However, we failed to collect enough data on HbA1c, which was extremely important for the assessment of diabetes.

## 5. Conclusions

From the short-term results, diabetes does not increase the risk of postoperative complications and surgical difficulty in AC patients who underwent pancreaticoduodenectomy. While from the long-term results, diabetes might adversely affect the recurrence of patients with AC after curative pancreaticoduodenectomy, leading to an increased risk of poor prognosis in early-stage patients. However, further studies involving a large sample size are needed to validate our results.

## Figures and Tables

**Table 1:** The baseline clinicopathologic characteristics of the diabetes group and the no diabetes group.

Characteristic	Total		Diabetes		No Diabetes		*p*-Value
	*n* = 266 (100.00%)		*n* = 32 (100.00%)		*n* = 234 (100.00%)		
Sex							0.44
Male	145	54.51%	20	62.50%	125	53.42%	
Female	121	45.49%	12	37.50%	109	46.58%	
Age							0.08
≤50	70	26.32%	4	12.50%	66	28.21%	
>50	196	73.68%	28	87.50%	168	71.79%	
Jaundice							0.15
No	68	25.56%	12	37.50%	56	23.93%	
Yes	198	74.44%	20	62.50%	178	76.07%	
Intraoperative transfusion							0.87
No	124	46.62%	14	43.75%	110	47.01%	
Yes	142	53.38%	18	56.25%	124	52.99%	
Tumor size							0.34
≤2 cm	125	46.99%	12	37.50%	113	48.29%	
>2 cm	141	53.01%	20	62.50%	121	51.71%	
Differentiation							0.37
Poor	104	39.10%	16	50.00%	88	37.61%	
Moderate	112	42.11%	13	40.63%	99	42.31%	
Well	49	18.42%	3	9.38%	46	19.66%	
Regional nodes examined							1.00
≤11	131	49.25%	16	50.00%	115	49.15%	
>12	135	50.75%	16	50.00%	119	50.85%	
T stage							0.19
T1	29	10.90%	2	6.25%	27	11.54%	
T2	104	39.10%	9	28.13%	95	40.60%	
T3	133	50.00%	21	65.63%	112	47.86%	
Lymph node metastasis							0.29
No	176	66.17%	18	56.25%	158	67.52%	
Yes	90	33.83%	14	43.75%	76	32.48%	
TNM stage							0.05
I	112	42.11%	10	31.25%	102	43.59%	
II	96	36.09%	9	28.13%	87	37.18%	
III	58	21.80%	13	40.63%	45	19.23%	
Blood vessel invasion							0.34
No	197	74.06%	21	65.63%	176	75.21%	
Yes	69	25.94%	11	34.38%	58	24.79%	
Adjuvant treatment							0.91
No	134	50.38%	15	46.88%	119	50.85%	
Yes	93	34.96%	12	37.50%	81	34.62%	
Unknown	39	14.66%	5	15.63%	34	14.53%	

**Table 2:** Short-term outcomes of ampullary adenocarcinoma patients between the diabetes group and the no diabetes group.

Safety	Total		Diabetes		No Diabetes		*p*-Value
	*n* = 266 (100.00%)		*n* = 32 (100.00%)		*n* = 234 (100.00%)		
Surgical time							0.07
≤6 h	183	68.80%	17	53.13%	166	70.94%	
>6 h	83	31.20%	15	46.88%	68	29.06%	
Blood transfusion							0.87
No	124	46.62%	14	43.75%	110	47.01%	
Yes	142	53.38%	18	56.25%	124	52.99%	
Postoperative complications							0.74
No	155	58.27%	20	62.50%	135	57.69%	
Yes	111	41.73%	12	37.50%	99	42.31%	
Postoperative bleeding							0.51
No	242	90.98%	28	87.50%	214	91.45%	
Yes	24	9.02%	4	12.50%	20	8.55%	
Infection							1.00
No	226	84.96%	27	84.38%	199	85.04%	
Yes	40	15.04%	5	15.63%	35	14.96%	
Gastroparesis							0.55
No	237	89.10%	30	93.75%	207	88.46%	
Yes	29	10.90%	2	6.25%	27	11.54%	
Fistula							0.41
No	225	84.59%	25	78.13%	200	85.47%	
Yes	41	15.41%	7	21.88%	34	14.53%	
Gastroenteric anastomotic fistula	3	1.13%	2	6.25%	1	0.43%	
Pancreatic fistula	15	5.65%	2	6.25%	13	5.56%	
Biliary fistula	23	8.65%	3	9.38%	20	8.55%	

**Table 3:** Postoperative recurrence between diabetes group and no diabetes group in ampullary adenocarcinoma patients.

Recurrence	Total		Diabetes		No Diabetes		*p*-Value
	*n* = 266 (100.00%)		*n* = 32 (100.00%)		*n* = 234 (100.00%)		
Recurrence							0.02
No	146	54.89%	11	34.38%	135	57.69%	
Yes	120	45.11%	21	65.63%	99	42.31%	
Locoregional recurrence							1.00
No	211	79.32%	25	78.13%	186	79.49%	
Yes	55	20.68%	7	21.88%	48	20.51%	
Systemic recurrence							<0.01
No	195	73.31%	16	50.00%	179	76.50%	
Yes	71	26.69%	16	50.00%	55	23.50%	
Liver metastasis							0.03
No	209	78.57%	20	62.50%	189	80.77%	
Yes	57	21.43%	12	37.50%	45	19.23%	
Lung/Bone/Other metastasis							1.00
No	260	97.74%	32	100.00%	228	97.44%	
Yes	6	2.26%	0	0.00%	6	2.56%	
Peritoneal seeding							0.11
No	256	96.24%	29	90.63%	227	97.01%	
Yes	10	3.76%	3	9.38%	7	2.99%	

**Table 4:** Univariate and multivariate analysis of short-term and long-term outcomes between the diabetes group and the no diabetes group.

Outcomes	Diabetes	No Diabetes	*p*-Value
	*n* = 32 (12.03%)	*n* = 234 (87.97%)	
Median overall survival time, months	28.5	34	
5-year survival rates/%	26.00%	47.10%	
Follow-up period	1~168	1~240	
No. of deaths	24	113	
Univariate HR (95% CI) for OS	1.698 [1.091–2.642]	1 (Reference)	0.019
Multivariate HR (95% CI) for OS	1.597 [1.005–2.537]	1 (Reference)	0.047
Univariate HR (95% CI) for RFS	1.897 [1.179–3.052]	1 (Reference)	0.008
Multivariate HR (95% CI) for RFS	1.768 [1.068–2.925]	1 (Reference)	0.027
No. of patients with PC	12	99	
Univariate OR (95% CI) for PC	0.818 [0.382–1.752]	1 (Reference)	0.605
Multivariate OR (95% CI) for PC	0.818 [0.382–1.752]	1 (Reference)	0.605
No. of patients with recurrence	21	99	
Univariate OR (95% CI) for recurrence	2.603 [1.200–5.246]	1 (Reference)	0.015
Multivariate OR (95% CI) for recurrence	2.384 [1.065–5.336]	1 (Reference)	0.035

OS: adjusted jaundice, lymph nodes metastasis, T stage, TNM stage; RFS: adjusted jaundice, tumor size, lymph nodes metastasis, T stage, TNM stage, blood vessel invasion; recurrence: adjusted lymph nodes metastasis, blood vessel invasion, T stage, TNM stage.

## Data Availability

The datasets used during the current study are available from the corresponding author upon reasonable request.

## Supplementary Materials

The following supporting information can be downloaded at: https://www.mdpi.com/article/10.3390/curroncol29100528/s1, Table S1. The multivariate Cox analysis of overall survival in ampullary adenocarcinoma patients; Table S2. The multivariate Cox analysis of recurrence free survival in ampullary adenocarcinoma patients.

## References

[1] Silva L.C., Arruda R.M., Botelho P.F.R., Taveira L.N., Giardina K.M., de Oliveira M.A., Dias J., Oliveira C.Z., Fava G., Guimarães D.P. (2020). Cap-assisted endoscopy increases ampulla of Vater visualization in high-risk patients. BMC Gastroenterol..

[2] Zimmermann C., Wolk S., Aust D.E., Meier F., Saeger H.D., Ehehalt F., Weitz J., Welsch T., Distler M. (2019). The pathohistological subtype strongly predicts survival in patients with ampullary carcinoma. Sci. Rep..

[3] Ishihara S., Horiguchi A., Miyakawa S., Endo I., Miyazaki M., Takada T. (2016). Biliary tract cancer registry in Japan from 2008 to 2013. J. Hepato-Biliary-Pancreat. Sci..

[4] Miyazaki M., Ohtsuka M., Miyakawa S., Nagino M., Yamamoto M., Kokudo N., Sano K., Endo I., Unno M., Chijiiwa K. (2015). Classification of biliary tract cancers established by the Japanese Society of Hepato-Biliary-Pancreatic Surgery: 3(rd) English edition. J. Hepato-Biliary-Pancreat. Sci..

[5] Zhang X., Sun C., Li Z., Wang T., Zhao L., Niu P., Guo C., Chen Y., Che X., Zhao D. (2021). Development and Validation of a New Lymph Node Ratio-Based Staging System for Ampullary Carcinoma After Curative Pancreaticoduodenectomy. Front. Oncol..

[6] Koontongkaew S. (2013). The tumor microenvironment contribution to development, growth, invasion and metastasis of head and neck squamous cell carcinomas. J. Cancer.

[7] Maskarinec G., Shvetsov Y.B., Conroy S.M., Haiman C.A., Setiawan V.W., Le Marchand L. (2019). Type 2 diabetes as a predictor of survival among breast cancer patients: The multiethnic cohort. Breast Cancer Res. Treat..

[8] Hsieh M.C., Zhang L., Velasco-Gonzalez C., Yi Y., Pareti L.A., Trapido E.J., Chen V.W., Wu X.C. (2022). Impact of diabetes and modifiable risk factors on pancreatic cancer survival in a population-based study after adjusting for clinical factors. Cancer Causes Control. CCC.

[9] Jing C., Wang Z., Fu X. (2020). Effect of diabetes mellitus on survival in patients with gallbladder Cancer: A systematic review and meta-analysis. BMC Cancer.

[10] Akhavan S., Ghahghaei-Nezamabadi A., Modaresgilani M., Mousavi A.S., Sepidarkish M., Tehranian A., Rezayof E. (2018). Impact of diabetes mellitus on epithelial ovarian cancer survival. BMC Cancer.

[11] Zheng X., Ma X., Deng H.Y., Zha P., Zhou J., Wang R.L., Jiang R. (2020). Diabetes mellitus and survival of esophageal cancer patients after esophagectomy: A systematic review and meta-analysis. Dis. Esophagus Off. J. Int. Soc. Dis. Esophagus.

[12] Yao W., Meng Y., Lu M., Fan W., Huang J., Li J., Zhu Z. (2018). Impact of type 2 diabetes mellitus on short-term and long-term outcomes of patients with esophageal squamous cell cancer undergoing resection: A propensity score analysis. Cancer Commun..

[13] Amshoff Y., Maskarinec G., Shvetsov Y.B., Raquinio P.H., Grandinetti A., Setiawan V.W., Haiman C.A., Le Marchand L. (2018). Type 2 diabetes and colorectal cancer survival: The multiethnic cohort. Int. J. Cancer.

[14] Yuan C., Zhang X., Babic A., Morales-Oyarvide V., Zhang Y., Smith-Warner S.A., Wu K., Wang M., Wolpin B.M., Meyerhardt J.A. (2021). Preexisting Type 2 Diabetes and Survival among Patients with Colorectal Cancer. Cancer Epidemiol. Biomark. Prev..

[15] Bi G., Yao G., Bian Y., Xue L., Zhang Y., Lu T., Fan H. (2020). The Effect of Diabetes Mellitus on Prognosis of Patients with Non-Small-Cell Lung Cancer: A Systematic Review and Meta-Analysis. Ann. Thorac. Cardiovasc. Surg. Off. J. Assoc. Thorac. Cardiovasc. Surg. Asia.

[16] Deng H.Y., Zheng X., Zha P., Peng L., Huang K.L., Qiu X.M. (2019). Diabetes mellitus and survival of non-small cell lung cancer patients after surgery: A comprehensive systematic review and meta-analysis. Thorac. Cancer.

[17] Slavchev S., Kornovski Y., Yordanov A., Ivanova Y., Kostov S., Slavcheva S. (2021). Survival in Advanced Epithelial Ovarian Cancer Associated with Cardiovascular Comorbidities and Type 2 Diabetes Mellitus. Curr. Oncol..

[18] Bitterman D.S., Winter K.A., Hong T.S., Fuchs C.S., Regine W.F., Abrams R.A., Safran H., Hoffman J.P., Benson A.B., Kasunic T. (2021). Impact of Diabetes and Insulin Use on Prognosis in Patients With Resected Pancreatic Cancer: An Ancillary Analysis of NRG Oncology RTOG 9704. Int. J. Radiat. Oncol. Biol. Phys..

[19] Qiang J.K., Sutradhar R., Giannakeas V., Bhatia D., Singh S., Lipscombe L.L. (2020). Impact of diabetes on colorectal cancer stage and mortality risk: A population-based cohort study. Diabetologia.

[20] Birch R.J., Downing A., Finan P.J., Howell S., Ajjan R.A., Morris E.J.A. (2021). Improving outcome prediction in individuals with colorectal cancer and diabetes by accurate assessment of vascular complications: Implications for clinical practice. Eur. J. Surg. Oncol. J. Eur. Soc. Surg. Oncol. Br. Assoc. Surg. Oncol..

[21] Yang I.P., Tsai H.L., Huang C.W., Lu C.Y., Miao Z.F., Chang S.F., Juo S.H., Wang J.Y. (2016). High blood sugar levels significantly impact the prognosis of colorectal cancer patients through down-regulation of microRNA-16 by targeting Myb and VEGFR2. Oncotarget.

[22] Chen L., Tao S.F., Zheng Y.X. (2006). Prognostic significance of vascular endothelial growth factor expression and microvessel density in carcinoma of ampulla of Vater. Hepatogastroenterology.

[23] Zheng Y., Wu C., Yang J., Zhao Y., Jia H., Xue M., Xu D., Yang F., Fu D., Wang C. (2020). Insulin-like growth factor 1-induced enolase 2 deacetylation by HDAC3 promotes metastasis of pancreatic cancer. Signal Transduct. Target. Ther..

[24] He L., Law P.T.Y., Wong C.K., Chan J.C.N., Chan P.K.S. (2017). Exendin-4 Exhibits Enhanced Anti-tumor Effects in Diabetic Mice. Sci. Rep..

[25] Lachmann G., von Haefen C., Wollersheim T., Spies C. (2017). Severe perioperative hyperglycemia attenuates postoperative monocytic function, basophil count and T cell activation. Minerva Anestesiol..

[26] Yachida S., Wood L.D., Suzuki M., Takai E., Totoki Y., Kato M., Luchini C., Arai Y., Nakamura H., Hama N. (2016). Genomic Sequencing Identifies ELF3 as a Driver of Ampullary Carcinoma. Cancer Cell..

[27] van Belle E., Cosenza A., Baptista S.B., Vincent F., Henderson J., Santos L., Ramos R., Pouillot C., Calé R., Cuisset T. (2020). Usefulness of Routine Fractional Flow Reserve for Clinical Management of Coronary 325 Artery Disease in Patients With Diabetes. JAMA Cardiol..

