# Supplementary material for: Impact of Diabetes on Short-Term and Long-Term Outcomes of Ampullary Adenocarcinoma Patients after Curative Pancreatoduodenectomy
Source: Curr Oncol. 2022 Sep 20;29(10):6724–34. doi: 10.3390/curroncol29100528 (PMC9600143; doi:10.3390/curroncol29100528)
Supplement: Supplementary file 1 [file curroncol-29-00528-s001.zip › curroncol-1897223-supplementary.pdf]

Supplementary Table S1. The multivariate Cox analysis of overall survival in ampullary adenocarcinoma patients.

Variables	HR	95% CI	*P-value*
Jaundice			
No		Reference	
Yes	1.499	0.979-2.296	0.063
T stage			
T1		Reference	
T2	1.272	0.646-2.506	0.487
T3	2.463	0.911-6.660	0.076
Lymph node metastasis			
No		Reference	
Yes	1.910	1.054-3.461	0.033
TNM stage			
Ⅰ		Reference	
Ⅱ	0.939	0.399-2.210	0.885
Ⅲ	0.747	0.230-2.424	0.627
Diabetes			
No		Reference	
Yes	1.597	1.005-2.537	0.047

Supplementary Table S2. The multivariate Cox analysis of recurrence free survival in ampullary adenocarcinoma patients.

Variables	HR	95% CI	*P-value*
Jaundice			
No		Reference	
Yes	1.563	0.973-2.511	0.065
T stage			
T1		Reference	
T2	1.395	0.635-3.065	0.408
T3	1.319	0.448-3.881	0.615
Lymph node metastasis			
No		Reference	
Yes	1.015	0.506-2.035	0.967
TNM stage			
Ⅰ		Reference	
Ⅱ	2.372	0.967-5.820	0.059

III	2.108	0.572-7.769	0.262
Tumor size			
≤2cm		Reference	
>2cm	1.229	0.831-1.817	0.302
Blood vessel invasion			
No		Reference	
Yes	1.376	0.850-2.228	0.194
Diabetes			
No		Reference	
Yes	1.768	1.068-2.925	0.027
